# Smartwatch-Derived Biomarkers Signal Pace of Biological Aging

**DOI:** 10.3390/dhi1010005

**Published:** 2026-07-09

**Authors:** Aminreza Khandan, Jian Sun, Sofi Majidi, Mohammad Dehghan Rouzi, Sona Mahrokhi Khoushehmehr, Helen Lavretsky, Gary W. Small, Linda M. Ercoli, Bijan Najafi

**Affiliations:** 1 Center for Advanced Surgical & Interventional Technology (CASIT), Department of Surgery, David Geffen School of Medicine, University of California, Los Angeles (UCLA), Los Angeles, CA 90095, USA; 2 Department of Psychiatry and Biobehavioral Sciences, David Geffen School of Medicine, University of California, Los Angeles (UCLA), Los Angeles, CA 90095, USA; 3 Department of Psychiatry and Biobehavioral Sciences, Semel Institute for Neuroscience and Human Behavior, David Geffen School of Medicine, University of California, Los Angeles (UCLA), Los Angeles, CA 90095, USA; 4 Department of Psychiatry and Behavioral Health, Hackensack Meridian School of Medicine, Nutley, NJ 07110, USA

**Keywords:** smartwatch, digital biomarkers, DunedinPACE, mobility, oxygen saturation, resting heart rate, older adults, wearable technology, biological aging

## Abstract

**Background:**

Biological aging varies substantially among individuals of the same chronological age, yet biological aging biomarkers require biospecimen collection and laboratory processing, limiting scalability for repeated monitoring.

**Objective:**

This study evaluated whether consumer smartwatch-derived digital biomarkers are associated with the pace of biological aging, quantified using DunedinPACE, a biomarker that measures biological aging rates.

**Methods:**

Baseline data were analyzed from the Healthy Minds for Life study within the Precision Aging Network. Among 1305 enrolled participants, 621 community-dwelling adults aged 50–79 years had both DunedinPACE and smartwatch data available. Smartwatch-derived biomarkers were grouped into mobility, cardiorespiratory physiology, sleep, and physiological stress domains. Associations with DunedinPACE were examined using Spearman correlations and adjusted linear regression models.

**Results:**

Significant associations were observed across all four domains. Daily step count showed the strongest mobility-domain association with DunedinPACE (r = −0.25, *p* < 0.01). Mean sleep peripheral oxygen saturation showed the strongest sleep-domain association (r = −0.22, *p* < 0.01). Resting heart rate showed the strongest physiological stress-domain association (r = 0.24, *p* < 0.01). Adjusted regression models supported these findings, with lower daily mobility, poorer sleep oxygenation and efficiency, greater wake after sleep onset, and higher resting heart rate showing the most consistent associations with higher DunedinPACE values.

**Conclusions:**

Smartwatch-derived biomarkers captured a multidomain profile of accelerated biological aging characterized by lower mobility, poorer sleep health, and elevated physiological stress. Consumer wearables can provide low-burden, real-world indicators of biological vulnerability, although longitudinal studies are needed before clinical use.

## 1. Introduction

Biological aging varies substantially among individuals of the same chronological age, yet estimating this variation requires biospecimen collection and laboratory infrastructure. Chronological age alone inadequately captures the biological processes underlying age-related vulnerability [1-4]. Individuals of the same age can differ markedly in their pace of biological aging, reflected in the rate of decline across cardiovascular, metabolic, renal, hepatic, pulmonary, and immune systems [2,3,5]. DNA methylation-based biomarkers have emerged as tools for quantifying this variation, evolving from early clocks estimating chronological age (Horvath, Hannum) to models incorporating mortality-related information (PhenoAge, GrimAge) [4,6-10]. More recently, DunedinPACE (Dunedin Pace of Aging Computed from the Epigenome) was developed to quantify the pace of aging rather than cumulative biological age, using longitudinal changes across multiple organ-system biomarkers distilled into a single methylation algorithm [11]. This distinction may offer greater clinical relevance, as pace-of-aging measures may be more sensitive to ongoing physiological decline and short-term intervention effects [8,12]. In cardiometabolic research, DunedinPACE has also been shown to predict future metabolic syndrome and track a greater burden of metabolic risk factors [13]. Importantly, DunedinPACE appears responsive to modifiable exposures—caloric restriction, physical activity, and cardiorespiratory fitness have all been linked to slower DunedinPACE trajectories [13-15]—suggesting it may be sensitive to intervention effects and relevant to clinical risk reduction. These findings position DunedinPACE as a potentially actionable biomarker; yet it is constrained by the infrastructure required to measure it.

Despite these advances, biological aging assessment remains largely confined to blood collection, laboratory processing, and analytic expertise, limiting scalability for population-level screening and repeated monitoring [16,17]. Most DNA methylation-based biomarkers, including DunedinPACE, rely on peripheral blood sampling and molecular assays that may be costly, time-intensive, and inaccessible in many real-world or resource-limited settings [11,16]. In addition, biomarker collection often occurs intermittently, constraining the ability to track short-term physiological changes or evaluate dynamic responses to behavioral and clinical interventions [12,16,17]. Accordingly, there is a growing need for scalable, low-burden approaches capable of identifying individuals with accelerated aging risk and enabling more frequent longitudinal assessment outside traditional research environments. Importantly, socioeconomic factors represent a critical upstream determinant of biological aging. Prior studies have demonstrated that indicators of neighborhood disadvantage, poverty, and structural racism are independently associated with accelerated DNA methylation-based aging, including DunedinPACE, with Black adults consistently showing higher pace-of-aging values compared with White adults, a disparity partly explained by differential exposure to social determinants of health. Monitoring biological aging in diverse, underserved communities, therefore, requires approaches that transcend the laboratory infrastructure constraints of molecular assays [18-20].

Current approaches to assessing aging-related risk are typically based on cross-sectional or episodic measurements, including clinic visits, laboratory testing, and one-time biospecimen collection, which provide only a limited snapshot of an individual’s physiological state [16,17]. However, biological aging is a dynamic and continuously evolving process shaped by fluctuations in physical activity, sleep quality, autonomic balance, metabolic stress, and environmental exposures over time [14,18,21]. Repeated or continuous monitoring may therefore offer a more sensitive, reliable, and ecologically valid representation of aging-related decline than intermittent measurements obtained under controlled settings [18,22]. Advances in wearable technology now create opportunities to longitudinally characterize real-world physiological function at scale, potentially complementing molecular biomarkers of aging with temporally rich behavioral data.

Commercially available smartwatches provide an increasingly accessible platform for continuous, real-world assessment of behavioral and physiological domains relevant to healthy aging, including mobility, sleep, cardiovascular function, and autonomic stress regulation [18,22]. Embedded accelerometers, photoplethysmography sensors, and pulse oximetry can estimate daily step count, resting heart rate, heart rate variability, sleep efficiency, and nocturnal oxygen saturation at scale [18,21,23,24]. Despite this potential, prior work has often relied on self-reported behaviors, research-grade devices, or isolated physiological domains, limiting translation into scalable clinical or community-based screening. Several studies have begun to bridge this gap. Fox et al. demonstrated that accelerometer-derived physical activity was associated with slower epigenetic aging in the Rhineland Study [23], while Ammous et al. replicated this finding in the Health and Retirement Study using objectively measured activity [24]. Similarly, Kawamura et al. linked cardiorespiratory fitness with DunedinPACE trajectories in older men [2,15], and Liu et al. showed that 12-year physical activity trajectories were associated with epigenetic age acceleration [14]. However, these prior investigations predominantly examined single physiological domains, relied on research-grade or non-wearable measurement tools, or were conducted at single sites, limiting generalizability and ecological validity [14,23]. Examining whether smartwatch-derived biomarkers are associated with DunedinPACE may determine whether consumer wearables can support pre-assessment or triage strategies for identifying individuals at higher aging-related risk.

In this study, we evaluated whether smartwatch-derived digital biomarkers are associated with the pace of biological aging, quantified using DunedinPACE. We monitored four clinically relevant domains: mobility, cardiorespiratory physiology, sleep, and physiological stress. We hypothesized that a smartwatch-derived profile characterized by lower mobility, poorer sleep health, and elevated physiological stress would be associated with higher DunedinPACE, indicating a faster pace of biological aging. By linking real-world wearable data with an epigenetic biomarker of aging, this study sought to assess whether consumer smartwatch biomarkers may provide low-burden indicators of biological vulnerability in community-dwelling older adults.

## 2. Materials and Methods

### 2.1. Participants and Data Collection

This study used baseline data from the Healthy Minds for Life (HML) multi-site study, a longitudinal digital biomarker cohort within the Precision Aging Network (PAN; “Precision Aging Network: Closing the Gap Between Cognitive Healthspan and Human Lifespan,” 5U19AG065169) [25]. The HML study characterizes inter-individual variability in the pace of aging and integrates multimodal assessments, including cognitive testing, neuroimaging, vascular imaging, biospecimens, and digital health data across multiple U.S. sites.

The present analysis represents a secondary, cross-sectional evaluation of an expanded cohort of 1305 community-dwelling adults aged 50–79 years without diagnosed dementia. Participants registered through the MindCrowd platform (https://mindcrowd.org/), a large web-based cognitive health study serving as the primary recruitment gateway for PAN, as well as through local recruitment at four academic centers: the University of Arizona, Johns Hopkins University, Emory University, and the University of Miami. The MindCrowd platform, including online screening and cognitive assessments, follows a separate protocol approved by the Western Institutional Review Board (WIRB; study number 1129241) and adheres to the Declaration of Helsinki.

Eligibility criteria included age ≥ 50 years, residence within 60 miles of a study site, English proficiency, and performance within the upper three quartiles on an age-, race/ethnicity-, and education-adjusted Paired Associates Test. The eligible participants have no known personal or family history of Alzheimer ’s disease and conform to MRI procedures. Exclusion criteria included prior diagnosis of dementia or major psychiatric disorders (e.g., schizophrenia or bipolar disorder) and contraindications to MRI. The cohort includes individuals from diverse racial and ethnic backgrounds, including non-Hispanic White, non-Hispanic Black, non-Hispanic Asian, and Hispanic/Latino participants.

Participants first completed online cognitive and health assessments in the MindCrowd platform, followed by in-person evaluations at one of the four study sites. These assessments include standardized neuropsychological testing, functional MRI, carotid ultrasound imaging, and multimodal biospecimen collection (blood, cerebrospinal fluid when available, and palmar sweat). Blood-derived DNA methylation data quantify the pace of aging by DunedinPACE. In addition, participants underwent continuous real-world monitoring using wearable devices (Garmin Ltd., Olathe, KS, USA), providing biomarkers of mobility, sleep, and physiological stress. The dataset enables evaluation of the relationship between real-world behavioral and physiological signals and molecular biomarkers of the pace of aging. The Western Copernicus Group Institutional Review Board (WCG; tracking number 20220776) approved the HML/PAN protocol (1328067), and all participants provided written informed consent.

### 2.2. Measurement of Biological Aging

DunedinPACE was computed from blood-derived DNA methylation data using the algorithm developed by Belsky et al. [11]. This algorithm applies elastic-net regression to cytosine-phosphate-guanine (CpG) methylation levels across 19 organ-system biomarkers to generate a single continuous pace-of-aging score. These biomarkers were assessed longitudinally over 20 years in the Dunedin cohort and distilled into a single-time-point methylation algorithm [11]. Higher values indicate faster biological aging. Of the 1305 enrolled participants, 982 had available DunedinPACE data and were included in the biological aging analysis (Figure 1).

Among 1305 enrolled participants, 982 had available DunedinPACE data, and 707 had available smartwatch data, resulting in an analytic sample of 621 participants with both measures. Participants were categorized by DunedinPACE into slow agers (*n* = 92), average agers (*n* = 418), and fast agers (*n* = 111). Smartwatch-derived biomarkers were grouped into four domains: mobility, cardiorespiratory physiology, sleep health, and physiological stress. The analysis evaluated associations between DunedinPACE and smartwatch-derived biomarkers across these domains.

### 2.3. Smartwatch-Derived Digital Biomarkers

Smartwatch-derived digital biomarkers were collected using Garmin wearable devices (Garmin Ltd., Olathe, KS, USA) through embedded triaxial accelerometers, photoplethysmography (PPG) sensors, and pulse oximetry-based estimates. Of the 1305 enrolled participants, 707 had available smartwatch data; combined with DunedinPACE availability, the analytic sample comprised 621 participants with both measures. These digital biomarkers were categorized into four domains: mobility, cardiorespiratory physiology, sleep, and physiological stress, and were defined in Table 1.

### 2.4. Statistical Analysis

Continuous variables were summarized as mean ± standard deviation, and categorical variables were summarized as frequency and percentage. Participants were categorized as slow, average, or fast agers based on DunedinPACE normative values (mean = 1.05, SD = 0.12) established by Belsky et al. [11] (Figure 1). Group differences in baseline characteristics were assessed using one-way analysis of variance or Kruskal–Wallis tests for continuous variables and chi-square or Fisher’s exact tests for categorical variables, as appropriate. DunedinPACE was analyzed as a continuous outcome, with higher values indicating a faster pace of biological aging. Associations between smartwatch-derived biomarkers and DunedinPACE were first evaluated using Spearman correlation coefficients. Pairwise Spearman intercorrelations among smartwatch-derived biomarkers were also examined and visualized using a heatmap to identify domain-level clustering. Linear regression models were then used to estimate the association between each smartwatch-derived biomarker and DunedinPACE. Regression coefficients (β) with 95% confidence intervals were reported. Adjusted models included race, sex, and BMI as covariates. Statistical significance was defined as a two-sided *p*-value < 0.05. Given the exploratory nature of this analysis and the number of comparisons performed, FDR correction was not applied; findings should be treated as hypothesis-generating and interpreted with caution pending replication in confirmatory studies. Given the cross-sectional design, all findings were interpreted as associations rather than causal effects. Analyses were conducted in Python 3.11 (Python Software Foundation, Wilmington, DE, USA) using SciPy 1.15, statsmodels 0.14, and scikit-learn 1.7.

## 3. Results

### 3.1. Participant Demographics

Participant characteristics stratified by biological aging group are shown in Table 2. The analytic sample included 621 participants, including 92 slow agers, 418 average agers, and 111 fast agers. Mean age (±SD) increased across biological aging groups, from 61.6 ± 7.7 years in slow agers to 64.4 ± 7.8 years in average agers and 64.9 ± 7.8 years in fast agers. This difference was statistically significant (*p* < 0.01). BMI also increased stepwise across groups, from 24.3 ± 3.9 kg/m^2^ in slow agers to 27.5 ± 6.3 kg/m^2^ in average agers and 30.4 ± 6.2 kg/m^2^ in fast agers (*p* < 0.01). Hispanic/Latino participants represented 16.3% of slow agers, 20.1% of average agers, and 19.8% of fast agers. Race distribution differed significantly across biological aging groups (*p* < 0.01). White participants represented 87% of slow agers, 75% of average agers, and 45% of fast agers. Black or African American participants represented 5% of slow agers, 17% of average agers, and 46% of fast agers. Other group differences were insignificant.

### 3.2. Associations Between Smartwatch-Derived Digital Biomarkers and DunedinPACE

Among mobility-related biomarkers, daily step count demonstrated the strongest association with DunedinPACE (r = −0.25, *p* < 0.001), with higher step counts associated with a slower pace of aging (Figures 2 and 3A). Most participants accumulated 3000–10,000 steps per day, while lower activity levels (<5000 steps/day) were more frequently associated with higher DunedinPACE values (>1.2). Sedentary behavior was positively associated with DunedinPACE, including sedentary percentage (r = 0.18, *p* < 0.001) and sedentary duration (r = 0.15, *p* < 0.001). Activity intensity biomarkers showed weaker but consistent inverse associations, including highly active duration (r = −0.12, *p* < 0.01), active duration (r = −0.10, *p* < 0.05), and moderate-vigorous activity (r = −0.08, *p* = 0.06, trend).

Within the cardiorespiratory physiology domain, resting heart rate showed a strong positive association with DunedinPACE (r = 0.29, *p* < 0.01), whereas SpO_2_ showed a negative association (r = −0.23, *p* < 0.01), indicating that elevated resting heart rate and reduced oxygen saturation were linked to faster biological aging (Figures 2 and 3B). Participants with SpO_2_ between 94–97% showed clustering around average DunedinPACE values, while those below 92% tended toward higher values. Resting heart rate ranged from 50 to 95 bpm and exhibited an approximately linear relationship with DunedinPACE, with values above 80 bpm more frequently associated with DunedinPACE above 1.0.

Within the sleep domain, sleep efficiency was inversely associated with DunedinPACE (r = −0.17, *p* < 0.01). Total sleep time (r = −0.12, *p* = 0.04) and time in bed (r = −0.11, *p* = 0.03) showed weaker negative associations, while deep sleep was not associated. Wake after sleep onset showed a positive association (r = 0.15, *p* < 0.01), suggesting fragmented sleep is linked to accelerated aging (Figures 2 and 3C).

Among physiological stress biomarkers, average stress (r = 0.13, *p* < 0.01) and high stress (r = 0.08, *p* = 0.04) were positively associated with DunedinPACE, indicating that higher stress burden is modestly related to faster aging (Figures 2 and 3D).

Pairwise intercorrelation analysis among selected smartwatch-derived biomarkers demonstrated clear domain-level clustering (Figure 4). Mobility biomarkers were strongly interrelated, including steps with active duration (r = 0.89, *p* < 0.01) and sedentary time (r = −0.82, *p* < 0.01). Sleep measures also showed expected clustering, with sleep efficiency strongly inversely associated with wake after sleep onset (r = −0.97, *p* < 0.01) and total sleep time strongly associated with total time in bed (r = 0.99, *p* < 0.01). Physiological stress-response measures were also related, with average stress correlated with high stress (r = 0.73, *p* < 0.01).

Adjusted linear regression models controlling for race/ethnicity are shown in Figure 4. In the mobility domain, the estimated β coefficients were approximately −0.01 for daily steps per 1000 steps, −0.16 for highly active duration, and −0.02 for active duration. In the cardiorespiratory physiology domain, β coefficients were approximately +0.003 for resting heart rate. In the sleep domain, β coefficients were approximately −0.01 for sleep efficiency, and +0.08 for wake after sleep onset (WASO). In the physiological stress domain, β coefficients were approximately close to 0.00 for average stress and high stress (Figure 5).

## 4. Discussion

We found that smartwatch-derived biomarkers captured a multidomain profile of accelerated biological aging, linking lower mobility, weaker cardiorespiratory physiology, poorer sleep health, and elevated physiological stress with higher DunedinPACE values. Specifically, lower step count and active duration, poorer sleep efficiency and nocturnal oxygenation, and higher resting heart rate and stress burden were directionally associated with higher DunedinPACE values. The intercorrelation analysis showed strong within-domain coherence and minimal overlap across clinical domains, suggesting each domain contributes independent physiological information to the wearable profile. Together, these findings suggest that everyday behavioral and physiological patterns captured passively by consumer wearables can reflect broader biological vulnerability in older adults. An important conceptual distinction underlies our study design. DunedinPACE is a relatively stable epigenetic biomarker that reflects the cumulative rate of biological aging and does not substantially change over days or weeks; longitudinal evidence indicates that it remains consistent across intervals of months to years in the absence of major lifestyle or clinical interventions. Our hypothesis was not that one week of smartwatch data directly measures biological aging. Rather, we posited that individuals experiencing accelerated biological aging exhibit characteristic habitual patterns of physical activity, sleep architecture, cardiovascular physiology, and autonomic regulation during daily life—patterns that are amenable to passive, continuous wearable-based capture. In this framing, smartwatch-derived metrics serve as digital phenotyping tools that index the behavioral and physiological manifestations of accelerated aging, while DunedinPACE quantifies the underlying biological rate. This conceptual distinction—between a stable biomarker of aging rate and continuous digital phenotyping reflecting aging-related physiological change—is important for interpretation. Ecologically valid, real-world monitoring complements the single-time-point nature of epigenetic assessment, and longitudinal wearable data collected over extended follow-up may eventually detect deviations from healthy aging trajectories before overt clinical decline, offering promise for scalable population-level screening. To our knowledge, this is the first multi-site cohort study to directly link DunedinPACE with smartwatch-derived real-world behavioral and physiological measures collected during activities of daily living across four geographically distinct study sites, spanning diverse racial, ethnic, and socioeconomic populations. Moreover, these biomarkers can be collected continuously in real-world settings, so they may complement traditional clinic-based assessments by capturing day-to-day functional status, sleep health, and autonomic strain outside the clinical environment.

Mobility-related biomarkers showed the strongest and most consistent associations with DunedinPACE. Lower step count, shorter active duration, and reduced highly active time were associated with faster biological aging, whereas greater sedentary time was positively associated. Mobility reflects integrated physiological function across multiple systems, including musculoskeletal, cardiovascular, metabolic, and neurologic health [26]. Prior studies have similarly reported that greater physical activity, including higher step counts and moderate-to-vigorous physical activity, and lower sedentary time are associated with more favorable epigenetic aging profiles [11,14,23,24]. Notably, moderate-to-vigorous physical activity showed only a nonsignificant trend toward lower DunedinPACE in the present cohort, which may reflect differences in cohort composition, activity-classification algorithms, and whether activity was measured objectively, self-reported, or summarized longitudinally. In this context, total daily movement and reduced sedentary behavior may serve as more sensitive markers. Effect sizes were modest, with daily steps (β = −0.01 per 1000 steps), active duration (β = −0.02 per hour/day), and highly active duration (β = −0.17 per hour/day) showing inverse associations, while moderate-to-vigorous activity and sedentary duration were near zero.

Cardiorespiratory physiology biomarkers showed the strongest associations with DunedinPACE. Resting heart rate was positively associated with faster biological aging (β = +0.003 per bpm), whereas lower SpO_2_ was associated with accelerated aging (β = −0.01), indicating the importance of cardiovascular function and nocturnal oxygenation. These findings are consistent with prior evidence linking autonomic imbalance and hypoxemia, and elevated resting heart rate to cardiometabolic risk and mortality [27], suggesting that these wearable-derived measures may capture fundamental physiological processes underlying biological aging.

Sleep biomarkers also showed meaningful associations with DunedinPACE. Reduced sleep efficiency was associated with faster biological aging. Additionally, WASO showed the strongest positive association (β = +0.08 per hour/night), whereas sleep efficiency showed small negative effects (β = −0.01). In contrast, after adjustment, total sleep time and time in bed duration were not linearly associated with DunedinPACE, suggesting that global sleep quality may be a more robust marker than consumer-derived sleep stage biomarkers. This finding is consistent with prior evidence linking sleep fragmentation [21] and sleep quality [28] with accelerated epigenetic aging, while supporting that smartwatch- derived sleep biomarkers could help identify individuals who warrant more detailed clinical or sleep evaluation.

Physiological stress-related biomarkers were modestly associated with biological aging. Average and high stress levels showed small positive associations with DunedinPACE, suggesting that higher physiological stress burden may relate to faster aging. However, the wearable-derived stress score likely reflects autonomic regulation rather than perceived psychological stress, and should therefore be interpreted cautiously as a preassessment marker rather than a clinical stress measure [29].

Collectively, the multi-domain wearable profile observed in faster agers—reduced physical activity, impaired sleep quality, reduced nocturnal oxygenation, and elevated autonomic stress—converges on biological mechanisms increasingly recognized as central regulators of aging trajectories. Physical inactivity and chronic sleep fragmentation have been shown to promote low-grade systemic inflammation and mitochondrial dysfunction, while sustained autonomic dysregulation and intermittent hypoxia have been shown to amplify oxidative stress, collectively contributing to accelerated cellular senescence. Several integrative signaling nodes—including the mechanistic target of rapamycin (mTOR), AMP-activated protein kinase (AMPK), and insulin/IGF-1 signaling—have been proposed as central regulators linking these cellular stressors to aging trajectories [30-32], Among these, mTOR functions as a key sensor of nutrient availability, energy status, and inflammatory signals, and its dysregulation has been linked to impaired autophagy, reduced mitochondrial biogenesis, and accelerated cellular senescence [33,34]. Physical inactivity and cardiometabolic stress have been associated with upregulation of mTOR-dependent pathways and downregulation of AMPK activity, whereas structured exercise and caloric restriction reverse this balance—a pattern consistent with the DunedinPACE associations identified in this study. These convergent pathways provide a plausible biological rationale for the association between wearable-derived multi-domain physiological burden and accelerated epigenetic aging. As illustrated in Figure 6, age-related decline in mobility and sleep quality has been linked to chronic low-grade inflammation and mitochondrial dysfunction [2,15,27,28], while autonomic dysregulation and intermittent hypoxia contribute to oxidative stress [23,35,36]. These processes converge on integrative nutrient-sensing pathways, including mTOR, AMPK, and insulin/IGF-1 signaling [33,34], which have been mechanistically linked to the pace of biological aging captured by DunedinPACE [11].

Race distribution differed significantly across biological aging groups (*p* < 0.001), with White participants more represented among slow agers and Black participants more represented among fast agers. This pattern is consistent with prior studies showing that DNA methylation-based aging biomarkers, including DunedinPACE, are influenced by social determinants of health [11,35,36]. Accordingly, race/ethnicity was included as a covariate in regression models, although residual confounding due to unmeasured socioeconomic, environmental, healthcare-access, and behavioral factors may remain. Future studies should incorporate these factors to better contextualize biological aging.

These findings suggest that several smartwatch-derived biomarkers associated with higher DunedinPACE represent potentially modifiable physiological risks. Because these biomarkers can be collected continuously and passively, they may serve as scalable preassessment signals to identify individuals with adverse physiologic profiles who may warrant more detailed evaluation. These wearable-derived signals should therefore be interpreted as preassessment markers rather than stand-alone clinical biomarkers. Longitudinal and interventional studies are needed to determine whether improvements in these domains are accompanied by favorable changes in DunedinPACE. Although cognition was not the primary outcome of this analysis, these findings may also inform future brain-health research because the identified wearable-derived domains overlap with physiological risk factors relevant to cognitive aging. Crucially, the wearable-derived biomarkers most strongly associated with accelerated biological aging in this study—including reduced daily step count, lower SpO_2_, impaired sleep efficiency, and elevated resting heart rate—are themselves targets of established behavioral and clinical interventions. Structured physical activity programs, continuous positive airway pressure therapy for sleep-disordered breathing, sleep hygiene interventions, and cardiovascular risk reduction strategies have each demonstrated efficacy in improving these specific parameters. Future interventional studies should therefore directly test whether wearable-guided improvements in these modifiable domains translate into measurable changes in DunedinPACE, thereby establishing whether consumer wearables can serve not only as passive risk indicators but as active monitoring tools within aging-focused clinical trials.

This study has several limitations. First, its cross-sectional design limits causal inference; longitudinal follow-up of the HML cohort is needed to determine whether baseline smartwatch-derived digital biomarkers predict subsequent changes in DunedinPACE and aging-related outcomes. Second, smartwatch-derived biomarkers should be interpreted as pre-assessment or triaging indicators rather than clinical diagnostics, as consumer devices are not clinical-grade and lack full validation against research standards such as polysomnography or clinical pulse oximetry. Third, residual confounding may influence the findings, and additional factors, including comorbidities, medication use, behavior, socioeconomic conditions, and environmental exposures, were not fully accounted for. The cohort also did not include adults >80 years old, a population with greater variability in health status. Fourth, the cohort exhibited a higher proportion of female participants (68%), which reflects well-documented patterns in aging research whereby women have longer life expectancy and greater engagement in health studies. Our regression models adjusted for sex; however, residual sex-specific differences in wearable-derived metrics and epigenetic aging trajectories may persist, and future studies with larger, balanced cohorts should systematically investigate sex-specific digital aging signatures. Fifth, the Garmin wearable used in this study was intentionally selected for its low cost and feasibility for deployment across geographically diverse PAN sites. While it captured several physiologically informative signals—including heart rate, SpO_2_, HRV-derived physiological stress, sleep, and physical activity—newer-generation wearables can additionally provide more granular heart rate variability indices, circadian rhythm characteristics, vascular stiffness estimates, skin temperature, and respiratory parameters. Future studies leveraging these richer sensor modalities may strengthen the wearable-biological aging association. Sixth, many variables not captured in this observational dataset—including smoking, diabetes, polypharmacy, and social determinants of health—are important determinants of both biological aging and the physiological parameters measured by smartwatches. Rather than serving as simple confounders, many of these factors likely lie on the causal pathway between lifestyle exposures and accelerated epigenetic aging. Properly disentangling mediation, confounding, and causal pathways requires dedicated causal inference analyses, including directed acyclic graph-based approaches, which are beyond the scope of the current observational study. Finally, intervention studies targeting physical activity, cardiorespiratory physiology, sleep quality, and cardiovascular fitness are needed to test whether improvements in digital biomarkers translate to changes in biological aging, alongside efforts to refine multi-biomarker risk models and validate them across diverse populations and clinical settings.

## 5. Conclusions

Smartwatch-derived digital biomarkers across mobility, cardiorespiratory physiology, sleep, and stress domains were associated with DunedinPACE in community-dwelling older adults, suggesting that consumer wearables may capture meaningful signals of biological aging in daily life. Daily step count, mean SpO_2_, and resting heart rate emerged as the strongest markers within their respective domains. From a translational perspective, smartwatch-derived measures may serve as low-burden triaging signals for accelerated aging, although longitudinal and interventional studies are needed before clinical integration. In addition, future deep learning models trained on continuous wearable data could estimate DunedinPACE directly, turning a one-time epigenetic snapshot into a continuously updated aging readout [37,38].

## Figures and Tables

**Figure 1.**
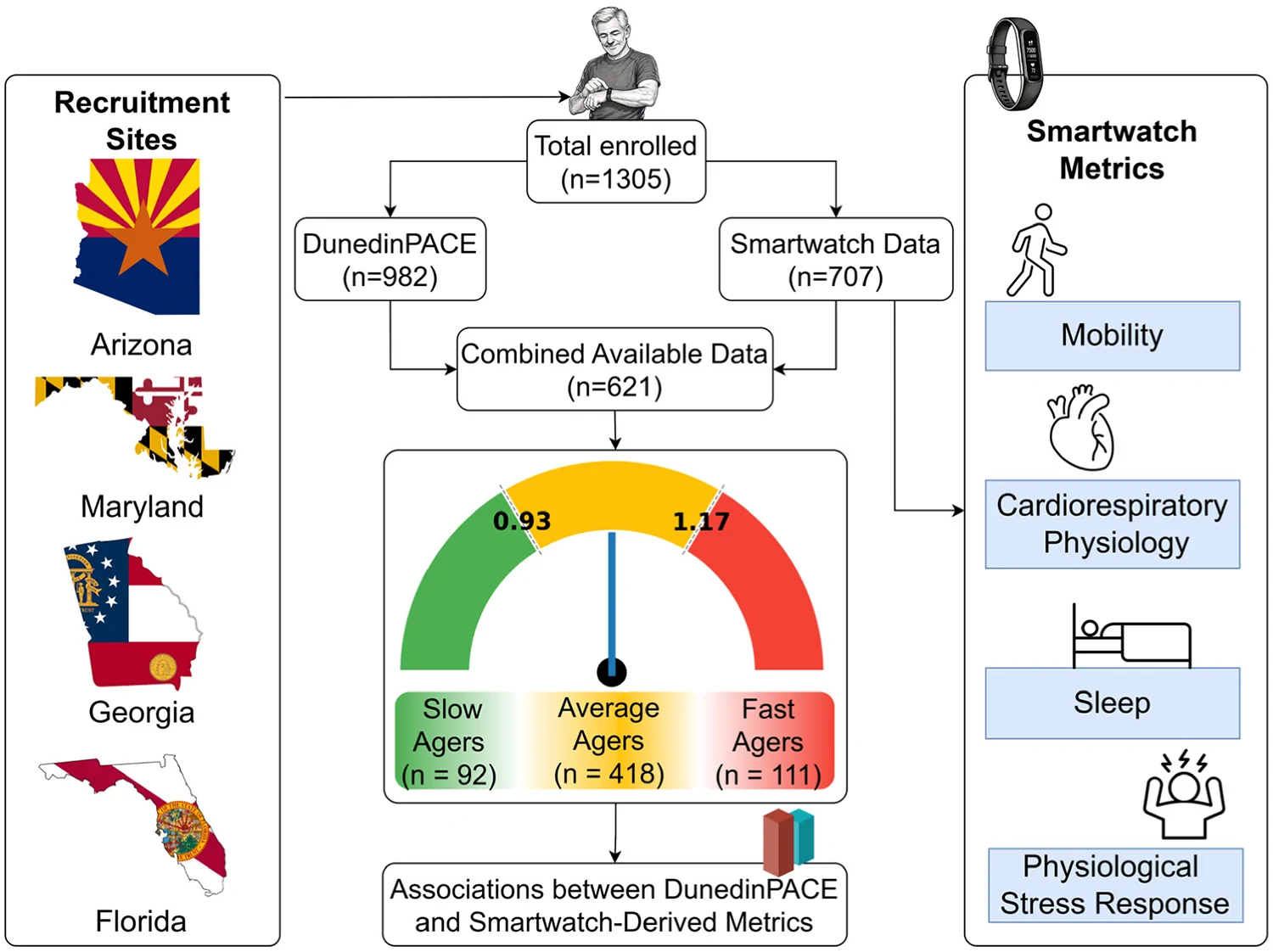
Study workflow and smartwatch-derived digital biomarker domains.

**Figure 2.**
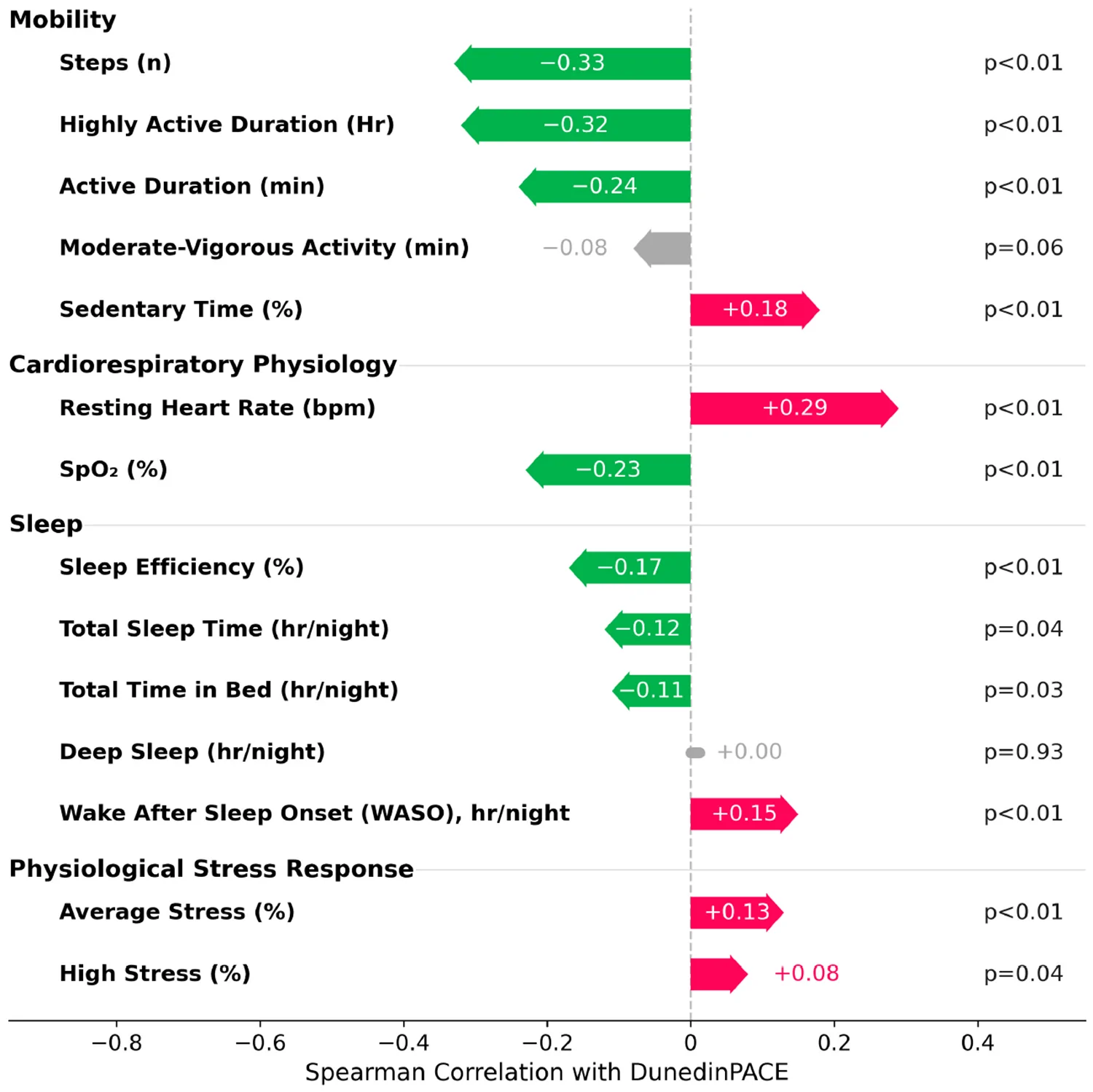
Spearman correlations between smartwatch-derived digital biomarkers and DunedinPACE across four domains: Mobility, Cardiorespiratory Physiology, Sleep, and Physiological Stress Response. Negative correlations (green) indicate slower biological aging, whereas positive correlations (red) indicate faster aging. Color intensity reflects correlation magnitude.

**Figure 3.**
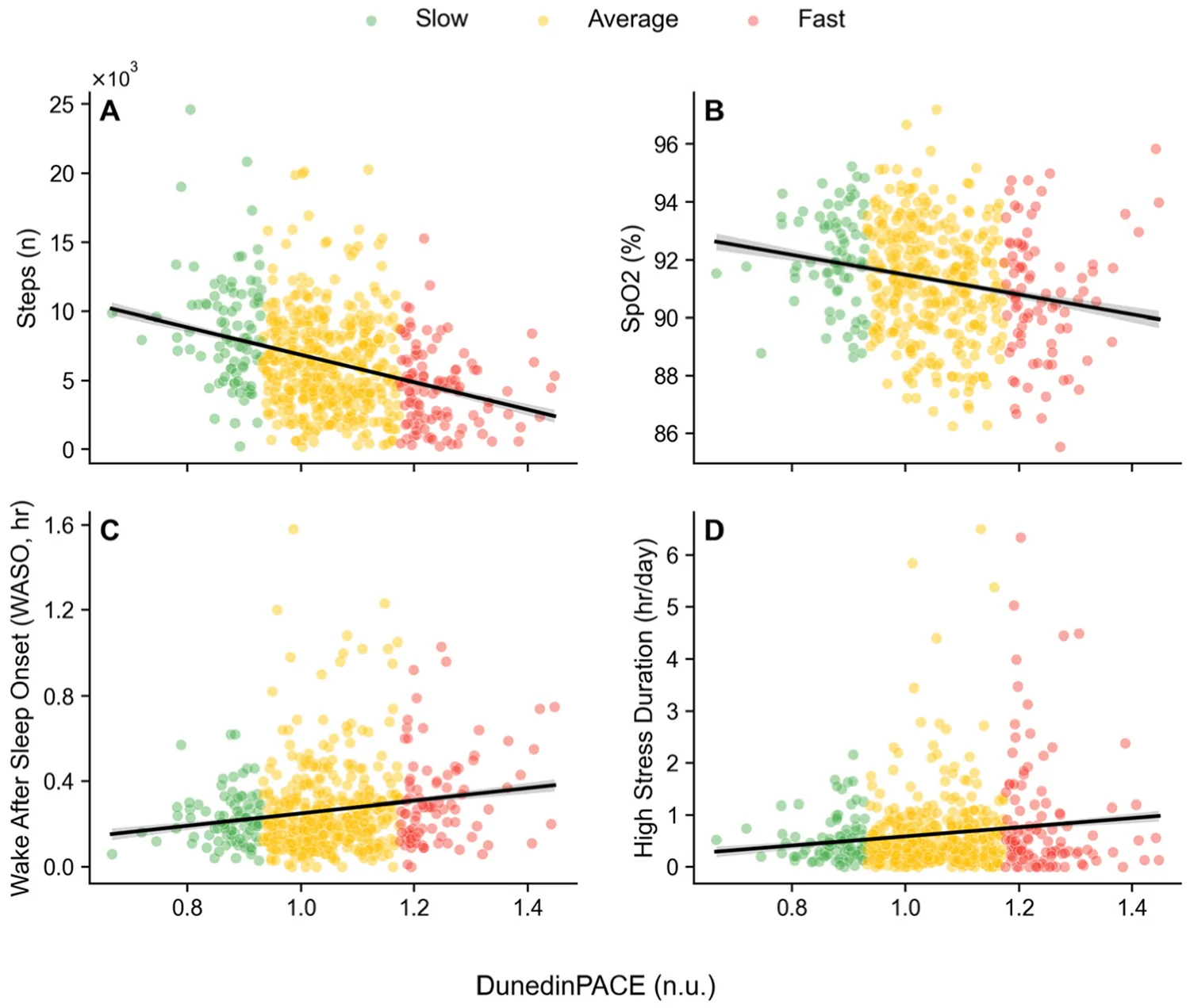
Scatter plots showing associations between the strongest digital biomarker predictors and DunedinPACE. Scatter plots display the relationships between (**A**) daily step count, (**B**) mean SpO_2_, (**C**) WASO, and (**D**) high stress duration with DunedinPACE in our dataset. Each point represents an individual participant. Solid lines indicate fitted linear regression lines with 95% confidence intervals (shaded regions). (**A**) Higher daily step counts were associated with slower pace of aging (r = −0.25, *p* < 0.01). (**B**) Higher mean peripheral oxygen saturation during sleep was associated with a slower pace of aging (r = −0.22, *p* < 0.01). (**C**) Higher WASO was associated with a faster pace of aging (r = 0.15, *p* < 0.01). (**D**) High Stress Duration was associated with a faster pace of aging (r = 0.08, *p* = 0.04).

**Figure 4.**
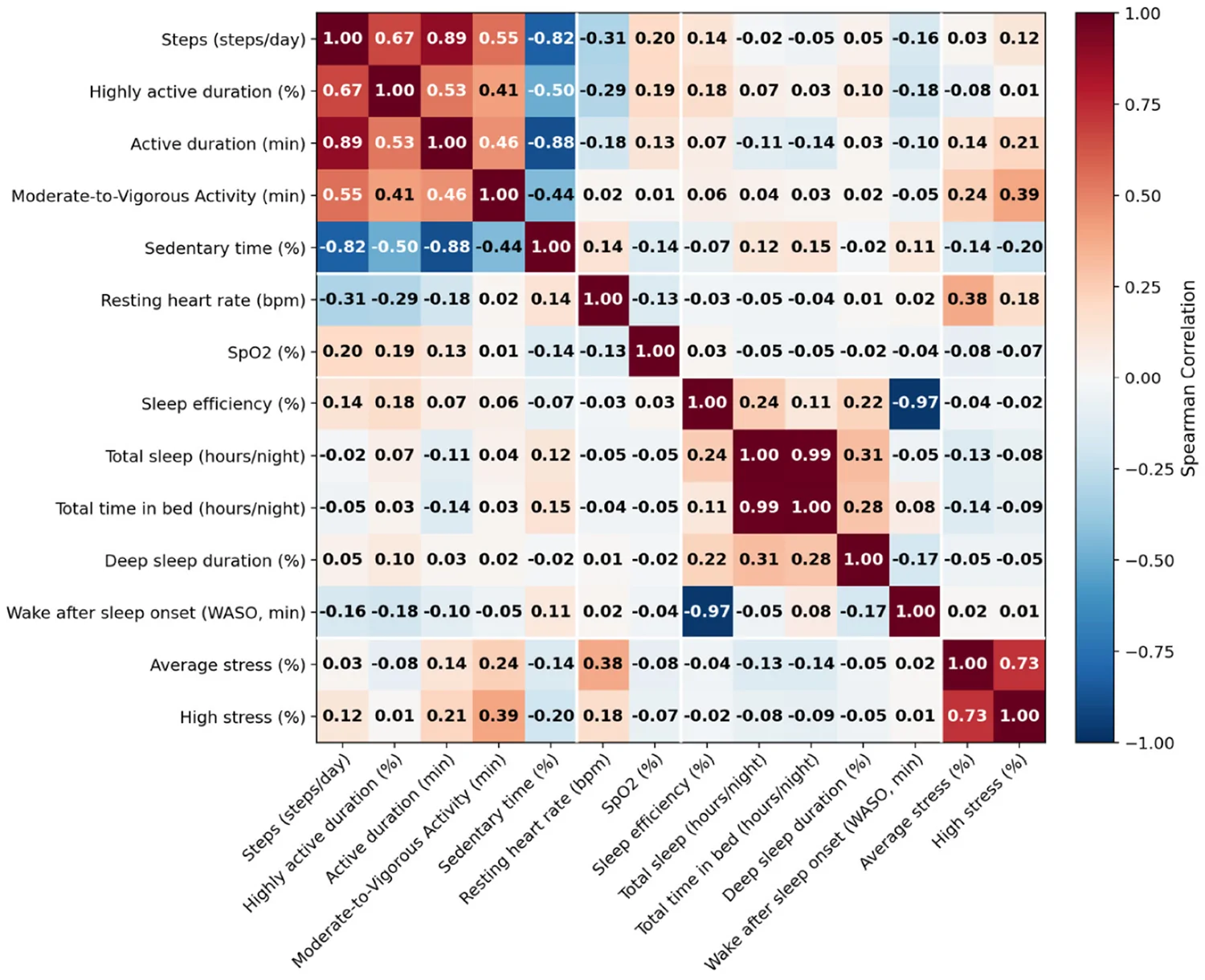
Intercorrelations among selected smartwatch-derived biomarkers. Pairwise Spearman correlations showed domain – level clustering among mobility, sleep, and physiological stress-response measures.

**Figure 5.**
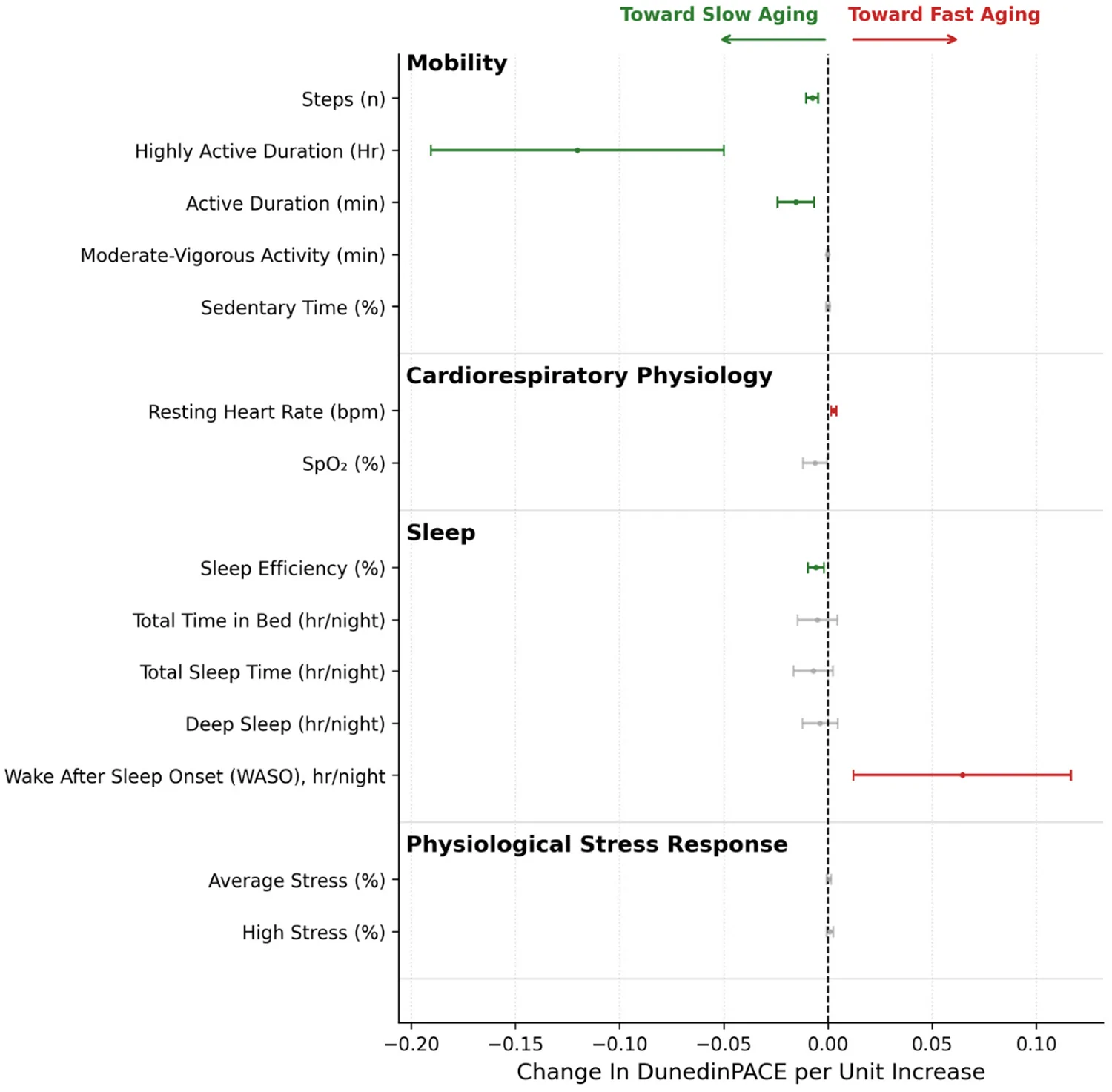
Association between smartwatch-derived digital biomarkers and biological aging (Dunedin-PACE). Forest plot showing regression coefficients (β) and 95% confidence intervals for the association between mobility, cardiorespiratory physiology, sleep, and physiological stress-related biomarkers and DunedinPACE. Negative values indicate associations with a slower pace of biological aging, whereas positive values indicate associations with a higher pace of aging. Models are adjusted for race/ethnicity. Green markers represent mobility, sleep variables, and SpO_2_ of cardiorespiratory physiology, and red markers represent physiological stress-related variables and resting heart rate of cardiorespiratory physiology; gray markers indicate non-significant associations. The vertical dashed line denotes no association (β = 0).

**Figure 6.**
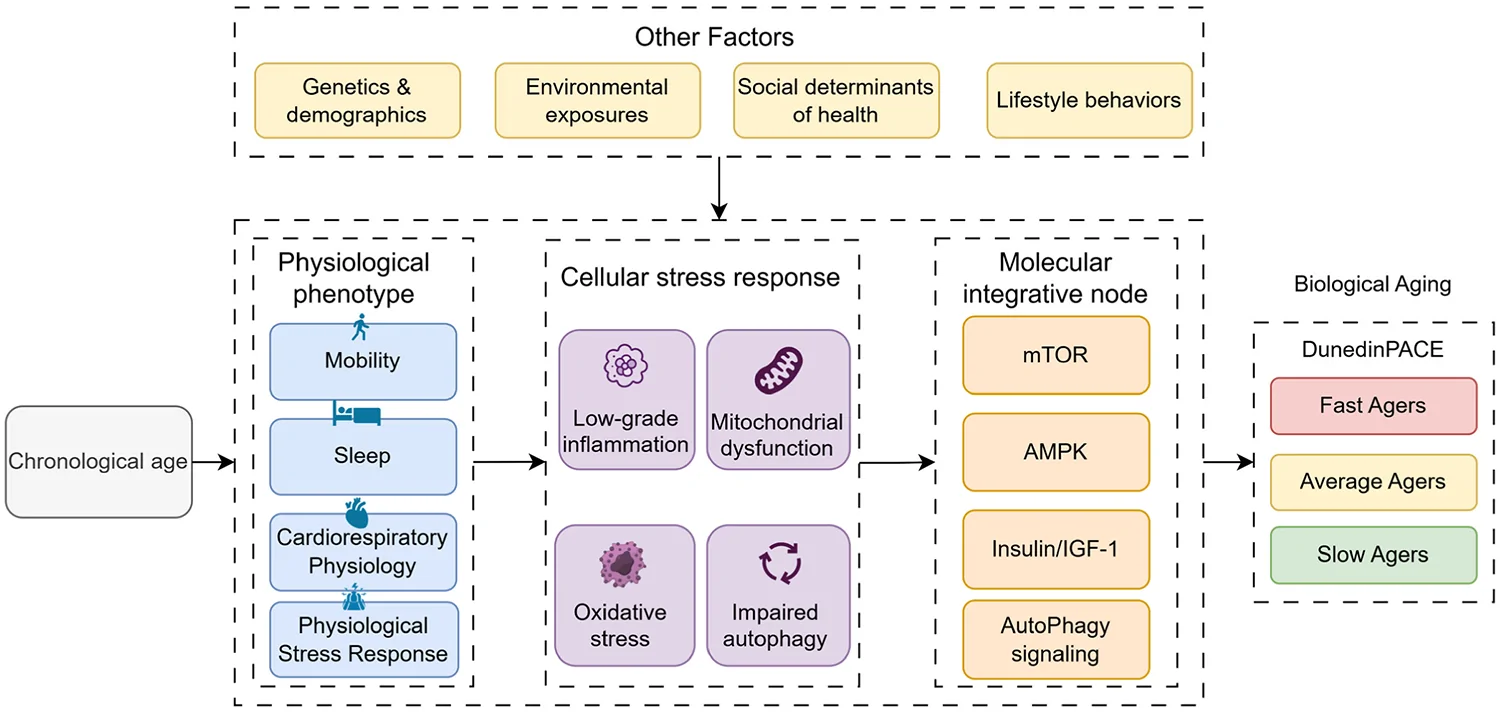
Proposed conceptual framework linking wearable–derived physiological phenotypes to biological aging. Chronological age influences four wearable-captured physiological domains (mobility, sleep, cardiorespiratory physiology, and physiological stress response) [1,26], which are further shaped by genetic, environmental, social, and behavioral factors [23,35,36]. These domains converge on cellular stress responses—low–grade inflammation, mitochondrial dysfunction, oxidative stress, and impaired autophagy—which are established hallmarks of biological aging [2,15,27,28]. These processes act through key molecular integrative nodes (mTOR, AMPK, insulin/IGF–1, and autophagy signaling) that regulate nutrient–sensing and longevity pathways, ultimately influencing the pace of biological aging as measured by DunedinPACE [11,33,34]. Solid arrows linking cellular stress responses and molecular integrative nodes reflect established mechanistic pathways supported by the cited literature [33,34]. Numbers in brackets refer to supporting references. This proposed framework is based on cross-sectional associations; it does not establish causal or temporal relationships between wearable-derived measures and biological aging. AMPK = AMP–activated protein kinase. IGF–1 = insulin–like growth factor 1. mTOR = mechanistic target of rapamycin.

**Table 1.** Definitions of Smartwatch-derived parameters for Mobility, Physiological Stress Response, Sleep, and Cardiorespiratory Physiology.

Category	Parameter (Unit)	Definition
Mobility	Steps (steps/day)	Total step count recorded during the monitoring period each day.
	Active duration (min)	Total duration spent in activities of moderate or higher intensity.
	Highly active duration (%)	Percentage of daily duration spent in high-intensity activity (e.g., running or speed walking).
	Moderate-to-Vigorous Activity (min)	Total duration spent in moderate or vigorous intensity activities.
	Sedentary time (%)	Percentage of daily duration spent with little to no activity.
Cardiorespiratory Physiology	Resting heart rate (bpm)	Minimum heart rate measured at rest, typically a 30 min rolling average, often occurring during sleep or sedentary periods.
	SpO_2_ (%)	Mean peripheral oxygen saturation during sleep (12–4 a.m.).
Sleep	Sleep efficiency (%)	Percentage of total time in bed spent asleep.
	Total time in bed (hours/night)	Total amount of time recorded from the start to the end of the sleep period.
	Total sleep (hours/night)	Total amount of time spent sleeping, excluding time awake.
	Deep sleep duration (%)	Proportion of deep sleep relative to the total sleep time.
	Wake after sleep onset (WASO, min)	Total time spent awake during the sleep period, after initial sleep onset.
Physiological Stress Response	Average stress (%)	Average level of stress over the total monitoring period during a day.
	High stress (%)	The percentage of the total monitoring period during which stress level measurements were in the high-stress range (76–100) in a day.

**Table 2.** Demographics of participants by rate of biological aging.

Variables	Slow Agers (*n* = 92)	Average Agers (*n* = 418)	Fast Agers (*n* = 111)	Total (*n* = 621)
**Age, years**	61.6 ± 7.7	64.4 ± 7.8	64.9 ± 7.8	64.1 ± 7.8
**Sex: *n* (%)**				
female	71 (77.2%)	276 (66.0%)	75 (67.6%)	422 (68.0%)
male	21 (22.8%)	142 (34.0%)	36 (32.4%)	199 (32.0%)
**BMI, kg/m^2^**	24.3 ± 3.9	27.8 ± 5.6	30.4 ± 6.2	27.6 ± 6.3
**Ethnicity**				
Hispanic/Latino, n (%)	15 (16.3%)	84 (20.1%)	22 (19.8%)	121 (19.5%)
Not Hispanic or Latino, n (%)	76 (82.6%)	319 (76.3%)	86 (77.5%)	481 (77.5%)
Not reported	1 (1.1)	15 (3.6%)	3 (2.7%)	19 (3.0%)
**Race, *n* (%)**				
White	80 (87%)	315 (75%)	50 (45%)	445 (72%)
Black or African American	5 (5%)	72 (17%)	51 (46%)	128 (21%)
Asian	0 (0%)	1 (0%)	1 (1%)	2 (0%)
American Indian or Alaska Native	0 (0%)	1 (0%)	0 (0%)	1 (0%)
Others	7 (8%)	29 (7%)	9 (8%)	46 (7%)

## Data Availability

Aggregated data generated and/or analyzed during this study are included in this article. Further enquiries can be directed to the corresponding author. De-identified, restricted data from this study will be available per request from the Precision Aging Network website after the first public data release in Spring 2026.
